# Peer effects of working capital management: Considering the moderating effect of knowledge flow

**DOI:** 10.3389/fpsyg.2022.1054349

**Published:** 2022-11-28

**Authors:** Mingyuan Zhao, Bingxin Ming, Yingqing Li, Junran Shi

**Affiliations:** Pan-Asia Business School, Yunnan Normal University, Kunming, China

**Keywords:** working capital, peer effect, knowledge flow, social cognition, Active Intermodal Matching theory

## Abstract

An important form of human learning and cognition is imitation. In environments where uncertainty is more incremental, imitation of peers is a natural response to uncertainty. While there are substantial literature documenting peer effects in other settings, the study of peer effects in working capital management is novel; little research exists on peer effects in working capital management and their impact mechanism. Using data of China’s listed firms from 2010 to 2021, we empirically demonstrate significant peer effects due to working capital management. Firstly, we find that the behavior of working capital management of firms in the same industry is positively related to a firm’s working capital management. We used peer firms’ target debt ratio as an instrumental variable to address potential endogeneity problem. Secondly, the moderating effects test shows that the positive relationship between the behavior of working capital management of firms in the same industry and a firm’s working capital management behavior is moderated by knowledge flow. Meanwhile, the peer effects in the high group of knowledge flow are greater than that of in the low group of knowledge flow. The study is based on the Active Intermodal Matching theory of psychology. It enriches the research findings on the moderating effect of peer effects and has important implications for policymaking to stimulate the economy.

## Introduction

The social psychology literature shows that the behavior of a subject is influenced by the behavior of other subjects in its group, which is a phenomenon known as the “peer effects.” Early evidence on psychological theories of behavioral imitation and social learning was presented by Bandura et al. (1961). Psychological group selection theory suggests that small groups of trust and cooperation are the basic, universal, form of sociality in the networks of usual cooperators. In the social network in which firms are embedded, the circle of peers is often one of the small groups. In dire straits, peer support seems to be clearly an advantage (Acedo-Carmona and Gomila, 2015). Psychological peer effects have received extensive attention in various fields including accounting and finance. The economic decisions of market participants are often influenced by each other, and the economic behavior of firms is not only based on the characteristics of their own resources and capabilities, but is also influenced by the behavior of peer firms (Crotty, 2003). Many studies have shown that there are significant peer effects in a firm’s investment behaviors (Brunner and Ostermaier, 2019), financing decision-making (Leary and Roberts, 2014), stock divisions (Adhikari and Agrawal, 2018) and tax avoidance (Gao et al., 2021). While there are substantial literature documenting peer effects in other settings, the study of peer effects in working capital management remains largely unexplored in the literature; little research exists on peer effects in working capital management and their impact mechanism. In this study, we investigate the role of peer effects in working capital management.

Many psychological theories have envisaged the combination of behavior parameters to explain the imitation behavior, but there is no way to put the brain’s consciousness into the parameter model, resulting in the lack of ability to explain the imitation behavior, so the AIM (Active Intermodal Matching) model was created. AIM theory proposes that the macro state of imitation consciousness can be explained by three parameters. The first parameter is A. Parameter A indicates information processing capacity. When the value of parameter A value is low, people have little ability to process information, their brains are in a coma, and people are unconscious. When the value of parameter A is high, people have great ability to process information and their brains are awake. The second parameter is I. The parameter I indicates the information source. When the value of parameter I is low, it indicates that the channel for information exchange is closed. At this time, people only pay attention to the information generated within the brain system. When the value of parameter I is high, it indicates that the information exchange channel is opened, and people pay attention to the information exchanged between the system and the outside world. The third parameter is M. The parameter M represents the chemical pattern that controls the brain. The significance of AIM model is that in the study of imitation consciousness, two groups of variables with different properties need to be involved each time. One is the variable of behaviorism method, and the other is the variable that can only be understood through human thinking (Hobson et al., 2000). The “idea” of an action can be awakened by perceiving the action, action imitation is therefore a natural by-product of action perception (Prinz et al., 2009). The AIM theory of psychology has held that imitation is intentional or goal-directed and the goal is to achieve a match between perceived and executed actions (Meltzoff, 2010).

Imitation is shown not only in the presentation of behavior, but also in psychological speculation and simulation (Meltzoff, 2007). When there is a shortage of funds for long-term investment activities such as fixed asset investment and innovation investment, firms can allocate their working capital investment to long-term investment projects to ensure that the projects are carried out smoothly and continuously because of the advantages of lower adjustment cost and easy realization to avoid the cost loss from frequent adjustment of fixed capital (Ding et al., 2013). Therefore, when peer firms change their working capital allocation decisions to cope with market shocks and gain sustainable development, the firms begin redesigning their working capital holdings, and this behavioral imitation is of two types: (1) information transmitted through previous communication channels between the prior adopter and the firm; and (2) information inferred from the results of the actions of industry pioneers (Reppenhagen, 2010). Research on the social influence of psychology suggests that there are a variety of different mechanisms that promote the diffusion of behavior (Bikhchandani et al., 1992). Some of mechanisms are economically rational, while others are driven by human psychology. The social norms interpretation of behavioral imitation suggests that when individuals identify with a social group, the behavior of others in that social group has a greater influence on the social norms of the observer. Such behavior can also be observed in firms. For example, the perceptions of subordinate managers can be influenced by their superiors’ evaluation styles.

Due to the existence of information asymmetry in the market, imitation of peers is a natural response to uncertainty (Lieberman and Asaba, 2006). Compared to developed Western countries, working capital management in China is relatively weak, and the influence of cultural embeddedness and informal institutions on economic activities is much more prevalent and profound in China than in the West, suggesting that Chinese firms are more likely to be influenced by other firms’ working capital management approaches when they undertake working capital management. Many supermarkets have seen the success of Wal-Mart in the U.S. in using the OPEM financing model to obtain funds for global expansion, and supermarkets of a certain size in China have copied the model, with some firms achieving success, such as Gome, and others failing, such as Suning. Do the similar working capital management patterns of these listed firms imply the existence of peer effects? If so, what the mechanisms work? The existing literature does not provide a reasonable explanation for these questions.

This study investigates the industry peer effects of firms’ working capital management using a sample of non-financial firms listed in Chinese A-shares from 2010 to 2021. This study finds that there is an industry peer effect in the working capital management of listed firms, and the working capital management decisions are significantly and positively influenced by firms in the same industry; the formation mechanism of the peer effects is empirically tested, and the moderating effect of knowledge flow is verified.

The possible marginal contributions of the study are: Firstly, based on the theory of AIM in psychology, the study expands the research on working capital management theory considering the moderating effect of knowledge flow. Secondly, although the international psychological community has recently begun to pay attention to the peer effect of accounting behavior, the literature is still small and limited to the existence test of peer effects of accounting behavior, and there is a lack of consensus and rigorous empirical test on the formation mechanism of peer effects. This study empirically demonstrates knowledge flow as a mechanism of peer effects in working capital management and conducts a rigorous empirical test of it, which will enrich the theoretical findings of peer effects.

## Theoretical background and hypotheses

### Peer effects mechanisms

Peer influence was defined as getting a balance between being oneself and conforming to group behavior (Hou et al., 2021). Working capital holding acts as a buffer against unexpected shocks to investment (Ben-Nasr, 2016), but there is an optimal threshold for its holding, and a reasonable amount of working capital can contribute to the improvement of corporate performance as well as the increase of value (Kieschnick et al., 2013). If the firm holds too much working capital, although it can meet the liquidity needs of the firm, it will reduce the return on assets of the firm because of its low return; if the firm holds too little working capital, the firm will incur the risk of capital chain breakage. The firm needs to make a trade-off between profitability and liquidity to determine the optimal level of working capital investment (Baños-Caballero et al., 2013).

Decision-making is a complicated process that includes various neural and psychological activities (Jin et al., 2017). How to determine the appropriate working capital holdings? Firms can make decisions either through rational calculations based on their own realities or by imitating the results of behaviors that other firms have implemented. Early research explained the convergence of corporate accounting behavior as an imitative strategy due to weak financial management skills or conservative managers facing decision ambiguity, and attributed this herding behavior of abandoning private information to the role of signaling mechanisms (Manski, 2000). In recent years, with the introduction of peer theory in social psychology, researchers have found that the influence among firms in the same industry is complex and that managers make merger and acquisition decisions (Shue, 2013), investment decisions (Foucault and Fresard, 2014; Leary and Roberts, 2014), and surplus management (Jackson et al., 2017), among other Financial decisions may be made either by directly imitating the results of decision making of peer firms or by updating the results of their own decision basis after learning knowledge of the financial behavior of other firms in the same industry.

In the face of economic downturn and uncertainty, imitating similar factors of peers can partially replace rational calculation factors, and the extent to which firms make decisions based on reference peers shows a positive relationship with the degree of uncertainty (Abrahamson and Rosenkopf, 1997). In the prospectuses published in China, many prospectuses benchmark the working capital management indicators of peers as a basis for judging the reasonableness of firms’ working capital holdings. Firms are often faced with uncertainty when making decisions. Logically, firms can reduce the uncertainty of their decisions by imitating the working capital management behavior of other peer firms (Haunschild, 1993). In inter-firm imitation activities, large firms that perform better and are more efficient are more likely to be the targets of imitation and are imitated to a greater extent. While firms are influenced by peer firms when making decisions about working capital holdings, they do not imitate mechanically, but decide on specific imitation strategies by observing information obtained from peer firms’ financial statements and other information sources. Accounting information disclosed by peer firms has incremental value and good predictive power for future revenues, working capital management ability, financial distress and bankruptcy risk, and their disclosures have positive spillover effects on users of accounting statements (Durnev and Mangen, 2020). The sales revenue and capital expenditure voluntarily disclosed by peer firms in accounting statements can help managers reading the statements to make more accurate estimates of market demand and supply conditions, gain timely insight into potential investment opportunities, reduce uncertainty about future cash flows of investment projects, and thus make better decisions (Bonsall IV et al., 2013).

Over-holding of working capital can lead to missed opportunities for mergers and acquisitions and lost opportunities to explore new markets, thus damaging the value of the firm, and reallocation of inefficiently allocated assets can improve the firm’s share price and its performance in the later years (Denis and Kruse, 2000). Since firms in the same industry face a similar market environment and are highly comparable, the experience of each other is more meaningful for the working capital decision, therefore, when making the decision on working capital holdings, listed firms are likely to obtain information from firms in the same industry and use the working capital management indexes of their peers as a reference, choose a higher working capital holding than their peers, and wait for a higher payout. The impact of peer firms’ financing decisions on other firms is more prominent than any other observable factor (Leary and Roberts, 2014). When firms observe that the working capital holdings of other peer firms can generally increase firm value, they will likely be more willing to imitate their peers by holding higher levels of working capital than peer firms; conversely, if they observe that the amount of working capital held by peer firms generally yields poorer performance, they will be less willing to imitate.

Another reason why firms are influenced by peer firms is spillover correlation, which means that prior adoption may change the net benefit of a later adopter’s decision. For example, prior adoption by an industry competitor will increase the likelihood that the firm will make a similar decision (Francis and Michas, 2013). Firms in the same industry with similar business activities are natural peers, as they face the same business environment, and perhaps, might be followed by the same media and financial analysts. Firms in the same industry are regularly compared by financial analysts and creditors. Since firms in the same industry are in a similar environment, they may choose the same similar accounting practices, such as identical mechanisms of earnings management (Kedia et al., 2015). Therefore, in the current study, hypothesis 1 is proposed, as follows:

*H1*: working capital management has peer effects; that is, the behavior of working capital management of firms in the same industry is positively related to a firm’s working capital management.

### The moderating effect of knowledge flow

The AIM model of psychology can explain that the imitation consciousness depends on the second parameter I and the combination of I and the other two parameters A and M. The brain is always receiving internal and external information. Only when it is aware of the objective existence of the external world can the brain repeatedly identify the reliability of external information and compare it with internal information to know what external information represents. However, what makes the brain aware of the existence of the external world? This can only be known through introspection and speculation (Hobson et al., 2000). The AIM model is beneficial for working capital management that firms’ manager should open the channels of knowledge flow. Knowledge flow can enhance the thinking ability of firm managers, enable them to better cope with the market competition of firms, and help them attain better performance for firms (Zhou and Caroline Bingxin, 2012).

Knowledge flow is one of the many “flows” such as capital flow and logistics, and it is particularly important for firms in the era of intellectual capital and human capital, and it has become a trend to integrate knowledge flow into enterprise supply chain management in order to realize firms’ value. Inter-firm competition leads to the flow of knowledge within the network, and imitative competition among neighboring firms has the most direct and intense impact on firm behavior and performance (Gnyawali and Madhavan, 2001). The former refers to imitation behavior in which firms are motivated to obtain information valuable for decision making through imitation and learning, while the latter refers to firms’ imitation of their competitors in response to competitive needs. Both types of model behavior are premised on the flow of knowledge. Knowledge flows are knowledge exchanges and interactions among members of social networks driven by motives such as interest or innovation, and mainly include knowledge transfer, sharing, integration, learning, and utilization (Khelladi et al., 2022). From the learning psychology perspective, it is an effective way for entrepreneurs to quickly acquire and accumulate experience through knowledge flow and to do so faster than through their own experiential learning (Baum et al., 2000). Knowledge flows include both symbolic and coded explicit knowledge flows such as joint ventures, alliances and contractual transactions, and tacit knowledge flows such as culturally embedded experiences and insights (Von Krogh and Geilinger, 2014). In the process of working capital management, explicit knowledge includes visualized or textualized knowledge such as various financial periodic reports, securities analysts’ reports, stock exchange prospectuses, and various inquiry letters. The flow of such knowledge is usually flowed and acquired by means of online materials. On the other hand, tacit knowledge is mainly hidden in the management experience, decision-making judgment and management methods of key management or technical personnel themselves, and its flow mainly relies on informal ways such as face-to-face communication. Knowledge always develops and improves according to the continuous development from tacit to explicit, generating new explicit knowledge from old explicit knowledge, and then turning to new tacit knowledge that is more conducive to enterprise innovation (Azan et al., 2017).

Essentially, the core of the working capital holding decision is the process of weighing the impact of working capital holding on the value creation and risk level of the firm, and the optimal level of working capital holding should be the level at which the firm’s value is maximized and the financial risk is minimized. In the real environment, due to differences in individual firm characteristics and external frictions, the firm’s working capital tends to deviate from the optimal level and is constantly adjusted. The determination of the optimal level is a difficult problem (Baños-Caballero et al., 2013). According to the knowledge flow theory, accounting information disclosed by peer firms can help firms preparing investment decisions to reduce investment uncertainty, especially when this firm is subject to common needs with the firm disclosing the information. The financial statements, management discussion and analysis disclosed in the annual reports of listed firms have high reference value for information user. The forward-looking letter of reports analyses the future business performance, development strategies and business policies of firms. The positive tone of management discussion and analysis in the reports may imply optimistic judgments about the future, which will change the information set of readers and affects the behavior of their investment decisions (Roychowdhury et al., 2019). Of course, if peer firms’ disclosures are truthful, their disclosures can generate positive externalities for the firms concerned, but negative externalities can also arise if peer firms misrepresent their performance.

Changes in resource operations and investment and financing decisions of peer competitors can put pressure on firms, forcing them to maintain a high level of innovation sensitivity, proactively absorb new knowledge, and adopt self-regulation and absorption of external knowledge to converge themselves with leading firms (Hoffmann et al., 2018). The flow of knowledge increases financial reporting transparency and reduces adverse selection behavior of corporate managers, thus reducing the cost of capital, enabling efficient resource allocation by increasing total factor productivity, keeping working capital in line with peers, and allocating more capital to higher-yielding projects such as innovation investments, thus enabling the follower firms to benefit from competition (Hashmi, 2013). The increasing quality of regulatory supervision of financial and non-financial information has improved the quality of information disclosure of peer firms and increased the comparability and relevance of information. This knowledge flow will enable firms to keep abreast of the capital allocation status of their peers and reduce the allocation of working capital with low yields in a timely manner. In addition, with the advancement of computer text analysis technology, the textual characteristics of non-financial information can be better portrayed, providing information users with incremental knowledge that was not available through previous channels, and providing feasible technical support for peers to learn from the accessibility of non-financial information disclosed mainly in textual form (Yuan et al., 2022). The availability of big data and sophisticated data analytics tools increases access to knowledge of peer working capital decisions. With the increase of knowledge, managers have reduced the investment decision-making errors caused by behavioral biases such as overconfidence, limited attention span, loss aversion and misjudgment. Sensible managers do not allocate capital to working capital that does not yield high returns (Baker and Wurgler, 2013). Based on this argument it can be reasonably inferred that the knowledge flow among peers can reduce managers’ uncertainty about their own firm’s growth opportunities, increase the allocation of higher-yielding long-term investments and reduce the allocation of lower-yielding working capital. Therefore, hypothesis 2 is proposed, as follows:

*H2*: The positive relationship between the behavior of working capital management of firms in the same industry and a firm’s working capital management behavior is moderated by knowledge flow.

## Study design

### Data source

The samples are taken from all A-share listed firms in China from 2010 to 2021. we sort data as the following criteria: (1) exclude insolvent firms; (2) exclude ST, ST* firms and financial firms; (3) exclude firms with total assets, total liabilities, operating revenue, and operating costs equal to 0 or missing. The financial data involved in the study are gathered from the CSMAR database. Since the firm management methods of Hong Kong, Macao and Taiwan firms and foreign-funded firms are different from those of domestic firms, we exclude these firms from the sample so that we can achieve the goal of an in-depth study on the heterogeneity of knowledge flow. When calculating the industry indicator variables, firms with less than 10 industries are also excluded to avoid their interference with the test results. At the same time, in order to avoid the influence of extreme variables on the study results, the continuous variables below the 1% and above the 99% quantile are subjected to winsorize. We obtain a maximum sample of 35,074 Chinese firms.

### Model specifications and measurement of variables

In order to verify the hypotheses proposed in Section “Peer Effects Mechanisms,” the following regression models are constructed as follows:


(1) $$\begin{array}{l} {\mathrm{DNWC}}_{\mathrm{it}}=\beta_{0}+\beta_{1}\;{\mathrm{peerNWC}}_{\mathrm{it}}\\ +\beta_{2}^{`}\mathrm{Cont}1_{\mathrm{it}}+\beta_{2}^{`}\mathrm{Cont}2_{\mathrm{it}}+\epsilon_{\mathrm{it}}\; \end{array}$$

The dependent variable, DNWC*_it_* is a proxy variable for working capital management as firm *i* in year *t*, and considering an incremental investment in working capital, DNWC = DNWC*_it_* – DNWC_*it*−1_, and divided by total assets to eliminate the size effect. Following the study of Iqbal and Zhuquan (2015), working capital is defined as the value of NWC calculated in Table 1 and divided by total assets to eliminate the size effect; another measure of working capital is the method of Baños-Caballero et al. (2014), NWC1 = Current Assets - Current liabilities, DNWC1 = DNWC1*_it_* – DNWC1_*it*−1_, In the baseline regression model, DNWC is used in the study; in the robustness test, DNWC1 is substituted in the study.

**Table 1:** Description of variables.

Variable	Abbreviation	Definition
Working capital	NWC	Current assets-current liabilities
	NWC1	(Net notes receivable + Net accounts receivable + Net prepayments + Net inventory) – (Notes payable + Accounts payable + Receipts in advance + Employee remuneration payable + Taxes payable)
Working capital of peer companies	peerNWC	(Industry total capital-Company’s own capital)/(N-1)
Knowledge flow	KF	The firm’s stock idiosyncratic return
Enterprise size	Size	ln(total assets)
Business age	Age	ln(Year of company establishment+1)
Business growth	Grow	Annual operating income growth rate
Balance sheet ratio	lev	The ratio of total debt to total assets
Tobin’s value	TobinQ	The ratio of the firm market value to total assets.
Cash holding ratio	cashR	The ratio of total debt to total assets
Net return on assets	ROA	The ratio of net profit to total assets
Peer company size	peersize	Average size of other companies in the same industry
Peer business age	peerage	Average age of other companies in the same industry
Peer business growth	peergrowth	Sales growth rate of peer companies
Peer balance sheet ratio	peerlev	Gearing ratio of peer companies
Tobin’s value	peerTobinQ	Tobin’s value of peer companies
Peer cash holding ratio	peercashR	Cash holding ratio of peer companies
Peer net return on assets	peerROA	Net return on assets of peer companies

The independent variable, peerNWC, is a proxy variable for working capital management of peer firms in the same industry as firm *i* in year *t*. We exclude firm i’s working capital management in computing the average frequency to avoid a mechanical correlation. We include firm-level control variables (Cont1) that are regarded as primary determinants of firms’ working capital management.

Referring to existing research (Kedia et al., 2015; Gao et al., 2021), this study controls other factors possibly influencing the working capital management including firm size, firm age, firm growth, gearing ratio, TobinQ, cash to total assets ratio, and return on assets (De Almeida and Eid 2014). We also include peer firm averages variables (Cont2) to control for the contextual effects and denote them by the prefix “peer” (Boone and White, 2015). $\epsilon_{\mathrm{it}}$ is the random error term of the model. In addition, industry and year dummy variables are also set in the study. The model focuses on the regression coefficient of working capital management of peer firms. If the working capital management behavior of peers directly affects the working capital management behavior of firms, β1 is significant positive, indicating the peer effects of working capital management. Table 1 provides details of the main variable.

### Statistical analysis

Descriptive statistics of all the dependent variable and independent variables involved in the regression model are illustrated in Table 2.

**Table 2:** Descriptive statistics for the sample.

Variable	*N*	Mean	Sd	Min	p25	p50	p75	Max
DNWC	30,589	0.014	0.233	−6.633	−0.020	0.011	0.047	33.081
peerNWC	30,581	0.012	0.021	−0.263	0.006	0.013	0.021	0.151
KF	21,926	0.077	0.243	−0.616	−0.023	0.024	0.117	1.074
Size	35,074	22.098	1.361	13.763	21.149	21.916	22.848	28.637
Age	35,075	2.870	0.362	0.000	2.639	2.944	3.135	4.159
lev	35,074	0.441	1.095	−0.195	0.249	0.410	0.578	178.345
ROA	35,074	0.037	0.702	−48.316	0.015	0.039	0.070	108.366
Growth	30,633	5.438	773.906	−2.733	−0.024	0.109	0.274	134,600.000
cashR	35,074	0.193	0.144	0.000	0.092	0.152	0.250	1.000
TobinQ	30,100	2.173	2.834	0.641	1.243	1.636	2.380	259.146
peersize	35,065	22.098	0.543	19.527	21.821	22.040	22.107	24.014
peerage	35,066	2.870	0.177	1.386	2.765	2.896	2.992	3.407
peerlev	35,065	0.441	0.083	0.128	0.386	0.416	0.455	0.929
peerROA	35,065	0.037	0.028	−0.155	0.024	0.034	0.048	0.182
peergrowth	30,625	5.205	157.010	−0.348	0.174	0.381	0.488	5,852.680
peercashR	35,065	0.193	0.049	0.087	0.172	0.178	0.202	0.605
peerTobinQ	30,093	1.855	0.564	0.821	1.536	1.759	2.089	13.003

Because the firms engage in different industries and have different sizes, their working capital management levels vary. The maximum, mean and minimal values of variables presented in Table 2 indicate this argument. At the same time, it can be found from the table that the mean value ratio of the incremental working capital allocation is 1.36%, which indicates that the working capital of Chinese listed firms is showed a slight increase, which is in line with the situation that the GDP growth rate of China has been declining in recent years; the mean value of the working capital allocation growth of peer firms is 1.24%, but the growth rate is lower than the mean value of individual firms. This initially indicates that the working capital allocation of individual firms is higher than that of the peer firms. In Table 2, we can also find that the average age of Chinese listed firms is 2.87 years; the average value of asset liability ratio is 44%; the average value of return on assets is 3.67%; the average value of sales revenue growth is 5.4%, which is very volatile and very unbalanced between firms despite extreme values are processed by winsorize mode; the average value of the ratio of cash to assets is 19%; the average value of TobinQ is 2.17.

## Result and discussion

### Testing of hypotheses

In this study, the main variables are analyzed for correlation. Except that the correlation coefficient between the net return on assets and the proportion of working capital holdings is 0.7881, the correlation coefficient between other variables is less than 0.5. This shows that there is little possibility of multicollinearity between other variables. Due to the length limitation of this research, the correlation coefficient between variables is not listed in the table.

In Table 3, Model 1 shows the results of the multiple regression analysis controlling for all control variables, and the correlation coefficient between the independent and dependent variables is 0.7810, which is significantly positive at the 1% level. In terms of economic significance, the behavior of working capital management of firms in the same industry is positively related to a firm’s working capital management, which statistically and empirically holds hypothesis H1, indicating the existence of a working capital allocation peer effect. Similar findings were reported by Machokoto et al. (2022), who found that the effect of peer influence is positive in working capital management.

**Table 3:** Peer effect regression results.

	Model 1	Model 2	Model 3
	Baseline regression	Changing dependent and independent variables	Firm level fixed effects
Variables	DNWC	DNWC1	DNWC
peerNWC	0.781***		0.784***
	(13.615)		(21.675)
peerNWC1		0.121**	
		(2.489)	
Size	−0.002**	0.007***	0.006***
	(−2.016)	(4.276)	(4.023)
Age	−0.010***	0.023***	−0.040***
	(−5.867)	(10.905)	(−4.187)
lev	0.004	−0.034**	−0.026***
	(0.413)	(−1.975)	(−4.274)
ROA	0.299***	0.456***	0.305***
	(6.818)	(8.138)	(43.160)
Growth	−0.000**	−0.000***	−0.000
	(−2.442)	(−2.855)	(−1.596)
cashR	−0.067***	0.107***	−0.104***
	(−8.247)	(10.721)	(−15.553)
TobinQ	−0.000	−0.000	0.001**
	(−0.102)	(−0.556)	(2.104)
peersize	0.009	−0.017**	−0.001
	(0.739)	(−2.047)	(−0.376)
peerage	−0.013	−0.011	−0.038*
	(−0.509)	(−0.332)	(−1.933)
peerlev	−0.014	−0.023	0.011
	(−0.605)	(−0.887)	(0.531)
peerROA	−0.125***	−0.119**	−0.122***
	(−4.205)	(−2.334)	(−4.316)
peergrowth	0.000	−0.000	0.000
	(0.672)	(−0.222)	(0.216)
peercashR	0.044	−0.199***	0.049
	(0.836)	(−4.614)	(1.535)
peerTobinQ	−0.000	0.003	−0.001
	(−0.172)	(1.130)	(−0.421)
Firm\year fixed effects	YES	YES	Only firm
Constant	−0.060	0.214	
	(−0.217)	(0.992)	
*N*	28,710	28,710	28,312
*R* ^2^	0.131	0.198	0.264

Robust t-statistics in parentheses, ***p < 0.01, **p < 0.05, *p < 0.1.

From the OLS analysis of control variables: Firstly, at the firm-level, firm size, firm growth, and cash to total assets ratio are negatively related to firms’ working capital allocation; firm age, and return on assets are positively related to firms’ working capital allocation; asset liability ratio, and TobinQ are not related to firms’ working capital allocation. Secondly, at the peer level, only the ROA of peer firms is negatively related to the working capital allocation of firms, while other variables are not related to the working capital allocation of firms. Although the OLS analysis does not control for endogeneity issues, it provides explicit premises about the effect of the findings and provides a minimum validation of the model.

## Robustness tests

### Changing the measuring method of the dependent variable and independent variables

To further verify the robustness of the baseline model, this section presents robustness tests conducted from the aspects of changing the measurement of working capital management and peer firms. In the robustness test, we replace DNWC, peerNWC with DNWC1 and peerNWC1, respectively. The regression results are shown in Model 2 of Table 3. In this case, the peer effects of working capital management are significant positive (*β*_1_ = 0.121, *p* < 0.05) at the 5% level, and the magnitudes are very robust.

### Firm-level fixed effects

In the baseline OLS model, there may be omitted variables not mentioned in the literature that affect working capital allocation, thus creating an endogeneity problem. Every firm has its specific attributes that may affect the independent or dependent variables, so we need to control the fixed effect at the firm level to avoid such situation. Hausman’s test for (fixed effects—random effects) is *χ*^2^(40) = 331.58 with value of *p* of 0.000. The statistical outcome suggests that a fixed effects model should be used. The fixed effect model eliminates those time-invariant characteristics to assess the net effect of the independent variable on the result variable. We substitute the baseline OLS model with the fixed effects model that controls for the effects of firm and time, and the outcomes are displayed in Model 3 of Table 3. The estimated coefficient is 0.7838, and the test results are still significant at the 1% level. This suggests that the core conclusion of our research, “a firm’s working capital management is positively influenced by peer effect of firms” still holds.

### Endogeneity test

From the logic of the interaction between the peer effects on the firm’s working capital allocation, there may be an endogeneity problem between them caused by mutual causality and sample selectivity bias. To reduce the endogeneity problem, we employ the instrumental variable method to test the endogeneity of explanatory variable.

Therefore, we refer to the relevant work of Denis and McKeon (2012) and Chang et al. (2014) and choose target debt ratio (peertr) as an instrumental variable because it satisfies the selection criteria of instrumental variables: Firstly, target debt ratio meets the exogenous assumption of instrumental variables, which excludes industry and market effects in the calculation of target debt ratio, and thus can accurately reflect the information of firm-level working capital management. Secondly, target debt ratio meets the correlation assumption of instrumental variables, where the target debt ratio is correlated with the peer firms’ working capital allocation. Table 4 shows the outcomes of instrumental variables tests. In Model 4, the outcomes of the first stage indicates that peertr is significantly negatively correlated with peerNWC. In Model 5, the second stage results indicate that the regression coefficient of peerNWC is significantly positively correlated with DNWC at the 1% level, and the F-statistic value is 930.078, which is greater than the empirical value of 10, indicating that the target debt ratio has a strong explanatory power for peerNWC and is unlikely to have a weak instrumental variable problem. Furthermore, after the exogeneity tests of the variable are carried out, the Durbin (score) chi2(1) is 4.36005(*p* = 0.0368), proving that the explanatory variable is endogenous. The outcomes of the study are Robust.

**Table 4:** Regression results with two-stage least square (2SLS).

	Model 4	Model 5
	First stage	Second stage
Variables	peerNWC	DNWC
peertr	−0.179***	
	(0.006)	
peerNWC		0.875***
		(0.204)
Size	0.000***	−0.003***
	(0.000)	(0.001)
Age	−0.001**	−0.010***
	(0.000)	(0.002)
lev	−0.001**	0.010***
	(0.001)	(0.003)
ROA	−0.001	0.296***
	(0.001)	(0.006)
Growth	0.000	−0.000
	(0.000)	(0.000)
cashR	−0.001	−0.065***
	(0.001)	(0.005)
TobinQ	0.000	−0.000
	(0.000)	(0.000)
peersize	0.044***	0.004
	(0.001)	(0.008)
peerage	−0.081***	0.004
	(0.004)	(0.033)
peerlev	−0.070***	−0.003
	(0.004)	(0.028)
peerROA	0.174***	−0.140***
	(0.004)	(0.046)
peergrowth	0.000	0.000
	(0.000)	(0.000)
peercashR	−0.205***	0.059
	(0.006)	(0.043)
peerTobinQ	0.003***	−0.001
	(0.000)	(0.002)
Year\industry fixed effects	YES	YES
Constant	−0.597***	−0.015
	(0.028)	(0.164)
*N*	28,140	28,140
*R* ^2^	0.571	0.116
First stage *F* statistic	930.078	

Standard errors in parentheses, ***p < 0.01, **p < 0.05, *p < 0.1.

### Moderating effect test

To test whether there is a moderating effect of knowledge flow (KF) between the allocation of working capital of the peer firms and the allocation of working capital of the firms, Equation (2) is developed in a bid to verify H2 as follows:


(2) $$\begin{array}{l} {\mathrm{DNWC}}_{\mathrm{it}}=\beta_{0}+\beta_{1}\;{\mathrm{peerNWC}}_{\mathrm{it}}+\beta_{2}{\mathrm{peerNWC}}_{\mathrm{it}}*KF\\ +\beta_{3}\;KF+\beta_{4}^{`}\;\mathrm{Cont}1_{\mathrm{it}}+\beta_{5}^{`}\mathrm{Cont}2_{\mathrm{it}}+\in_{\mathrm{it}}\; \end{array}$$

Where, *i* and *t* represent firm, year, respectively. ∈ denotes residuals. DNWC, peerNWC and Controls are in keeping with how the variables are defined in the previous baseline regression. The moderating variable, KF, is a proxy variable for knowledge flow. Knowledge is inherently intangible and knowledge flow is in a constant state of change, so measuring the degree of knowledge flow becomes very difficult (Nissen, 2019), especially for tacit knowledge. The number of citations or citations of patent and non-IP documents are often used by scholars to measure knowledge flow because of their tangible and traceable knowledge flow relationship, but they are mainly used to reflect the flow trajectory of explicit knowledge and cannot reflect the flow of tacit knowledge (Wu and Mathews, 2012). Further, some scholars compute the number of clicks on patents, brands and publications of Baidu and Google search engine firms to reflect the firms’ knowledge flow. However, due to the current trend of mobile applications to replace computer search engines, the accuracy of this indicator is questioned. Regardless of the above-mentioned methods, it is impossible to make accurate measurement of knowledge flow intensity. It is especially important to choose appropriate methods in the face of different application research purposes and scenarios, especially to combine subjective and objective methods to jointly evaluate Knowledge Flow intensity from multiple perspectives. According to the efficient market theory, all information and knowledge flow are fully or partially reflected in the stock price, and the stock idiosyncratic return is a suitable metric for knowledge flow. Referring to the study of Liu et al. (2019), the intensity of the KF is computed as follows:


(3) $${KF}_{\mathrm{it}}=\alpha +\beta_{ijt}^{M}\left(\gamma_{t}^{M}-rf_{t}\right)+\beta_{ijt}^{ind}\left(\gamma_{ijt}^{\mathrm{peer}}-{rf}_{t}\right)+\varepsilon_{ijt}$$

where the indices *i*, *j*, and *t* correspond to firm, region, and month, respectively. $\gamma^{M}$ denotes the market rate of return, $\gamma^{\mathrm{peer}}$ denotes the peer firms’ stock yield. $rf_{t}$ denotes the risk-free rate of return. The specific computing steps are as follows: Firstly, at the beginning of each year, the monthly data of the industry for the previous 36 months are used to regress Equation (3), and the corresponding coefficient values are calculated. The regression coefficients at the beginning of the year are used to calculate the expected value of corporate stock returns for each month, and the actual stock returns for each month are subtracted from the expected stock returns to obtain the volatility of corporate stock returns for each month, i.e., the firm stock idiosyncratic returns for each month in the year. Secondly, the arithmetic mean of the firm’s stock idiosyncratic return for each month of the year is calculated to obtain the annual firms’ stock idiosyncratic return. Thirdly, the average annual stock idiosyncratic return of other firms in the same industry is calculated and used as a proxy variable for knowledge flow in the study.

Model 6 is developed to test the hypothesis (H2). We added knowledge flow (KF) as a moderator variable to our Model 6. The coefficient of KF is also positively significant with the peerNWC in Model 6 (*t* = 5.071, *p* < 0.01). When we implement our full interaction models peerNWC × KF, the coefficient is positively significant with the peerNWC in Model 6 and supports H2 (*t* = 3.316, *p* < 0.01). The results confirm that knowledge flow acts as a moderator. The economic implications of this outcome indicate that knowledge flow has an enhanced relationship between the behavior of working capital management of firms in the same industry and a firm’s working capital management behavior. In other words, the stronger the knowledge mobility is, the more firms and peers communicate is, the greater the amount of information acquired is, and the more significant the peer effects is.

Moreover, to further explain the moderating effect of knowledge flows, the research samples are divided into two subsamples, including the low group of knowledge flow and the high group of knowledge flow. We define the sample group with knowledge flow greater than the industry median as the high group of knowledge flow and the sample group with knowledge flow lower than the industry median as the low group of knowledge flow. The value of the high group is coded “1,” otherwise it is coded “0.” This study still uses the empirical model constructed by Equation (2), and the regression outcomes are reported in Table 5.

**Table 5:** Moderating effect of knowledge flow.

	Model 6	Model 7	Model 8
	All samplesDNWC	Low groupDNWC	High groupDNWC
peerNWC	0.396^***^	0.231^***^	0.524^***^
	(5.071)	(2.831)	(6.642)
peerNWC*KF	1.038^***^	0.843^***^	0.985^***^
	(3.316)	(4.219)	(3.431)
KF	−0.014^**^		
	(−2.041)		
Size	−0.002^***^	−0.001	−0.002^***^
	(−3.582)	(−1.450)	(−2.915)
Age	−0.011^***^	−0.011^***^	−0.011^***^
	(−7.112)	(−4.400)	(−4.422)
lev	−0.004	−0.005	−0.003
	(−0.703)	(−0.991)	(−0.556)
ROA	0.250^***^	0.274^***^	0.217^***^
	(11.346)	(22.661)	(16.275)
Growth	−0.000^***^	−0.000^*^	0.000^***^
	(−2.593)	(−1.798)	(5.046)
cashR	−0.067^***^	−0.071^***^	−0.064^***^
	(−11.364)	(−10.382)	(−9.432)
TobinQ	0.001	0.000	0.003^***^
	(1.095)	(1.002)	(6.303)
peersize	−0.014	−0.021^**^	−0.004
	(−1.506)	(−2.045)	(−0.397)
peerage	0.014	−0.008	−0.014
	(0.439)	(−0.182)	(−0.332)
peerlev	−0.003	−0.024	−0.006
	(−0.101)	(−0.686)	(−0.169)
peerROA	−0.037^***^	−0.035^***^	−0.048^***^
	(−2.169)	(−1.857)	(−3.216)
peergrowth	0.000	−0.000	0.000
	(0.143)	(−1.055)	(1.111)
peercashR	−0.038	−0.114^**^	0.018
	(−0.954)	(−2.295)	(0.388)
peerTobinQ	−0.000	−0.000	0.001
	(−0.090)	(−0.127)	(0.389)
_cons	0.374	0.577^**^	0.218
	(1.553)	(2.142)	(0.820)
Year\industry fixed effect	YES	YES	YES
*N*	17,758	8,821	8,872
Adj. *R*^2^	0.084	0.099	0.077

T statistics in parentheses, *p < 0.1, **p < 0.05, ***p < 0.01.

In the two subsamples of low group and high group, the regression results can be seen in Model 7 and Model 8 of Table 5: in the low group of knowledge flow, the coefficient of moderating effect is 0.843, which is significant at the level under 1%; in the high group of knowledge flow, the coefficient of peer effects is 0.985, which is significant at the level under 1%. In the study, after doing a mean comparison test for the difference in coefficients between groups, the results show that the difference in means is significant at the 1% level. This indicates that the difference in coefficients between the two groups is comparable. This also suggests that the moderating effect of knowledge flow in the high group of knowledge flow are greater than that of in the low group of knowledge flow, explaining the paradox of why the full sample knowledge flow has a negative effect on the peer effects. In the high group of knowledge flow, the correlation coefficient of ROA for the financial characteristics of the group of firms is significantly different from zero, indicating that a firm adjusted their working capital management by imitating and learning the financial characteristics of peer firms.

The peer effects of working capital management consist of three components: subject, the imitated behavior and environment. Among them, the imitation ability of imitators in the subject determines the effective implementation of the imitated behavior. Firstly, from the perspective of the imitated, their high working capital allocation ratio indicates a lack of confidence in the future and an increased incentive to prevent saving; a low working capital allocation ratio indicates that the firm mainly relies on long-term investment layout to support its development and is confident in the future. Therefore, the working capital allocation behavior of industry leaders is significant for industry followers and can attract them to imitate it. For industry followers, the uncertainty of economic policies can significantly increase the loan cost of firms in the process of GDP downturn. By imitating and learning to adjust their working capital and long-term capital structure, firms can not only reduce the financing cost, but also effectively reduce the financial risk caused by investment misjudgment.

Secondly, in terms of imitators, it is generally small firms that imitate large firms, and few large firms imitate small firms. The imitation behavior reflects management’s risk preference in the investment process (Zhou and Xu, 2019). The lower the risk-taking level of management is, the weaker the risk appetite will be, and the more liquid assets such as trading financial assets will be allocated. The more stable the working capital decisions of firms become, the stronger the need for managers to refer to similar decisions of peer firms. The more obvious the peer effect of working capital management is, the stronger the ability of imitating other firms to allocate assets is.

Thirdly, working capital allocation decisions are not only determined by the internal resources and capabilities of firms, but also closely related to the external macro environment. The environment of knowledge flow will affect the firms’ ability to adjust long- and short-term capital structure. In addition, the rise and fall of the industry will also affect the demand for working capital of firms, and thus affect the willingness and direction of firms’ working capital adjustment.

## Conclusion

Based on the Active Intermodal Matching theory of psychology, this study investigates the peer effects of working capital management using data from listed firms in China from 2010 to 2021 to draw the following conclusions: Firstly, there is a peer effect in firms’ working capital allocation. Secondly, the amount of corporate working capital allocation increases with the increase of peer firms’ working capital allocation. Thirdly, the peer effect of working capital works through the channel of knowledge flow, and the peer effects in the high group of knowledge flow are greater than that of in the low group of knowledge flow.

### Theoretical implications

The study has important theoretical contributions. Firstly, it verifies the existence of the peer effects of working capital allocation decisions and analyzes the mechanism of the peer effects of working capital decisions qualitatively through the channel of knowledge flow. It is different from most of the prior study that only analyzes the peer effect through the mechanism of imitation and learning. It answers the question, “What channels do firms learn from their peers?”

Secondly, it conducts a rigorous empirical test of knowledge flow as a mechanism of peer effects in working capital management, which will enrich the international research on accounting behavior and provide a reference for other studies on the peer effects of accounting behavior.

### Practical implications

The study also has important practical implications. Firstly, it reveals the facts that for firm practitioners, adjusting the amount of working capital allocation is an important means to hedge against economic uncertainty; and imitating the behavior of peer firms can reduce the uncertainty of decision making. However, when the competition in the same industry becomes more and more intense, it is easy to form vicious competition and produce over-investment or under-investment, therefore, firms should focus on considering the behavior of competitors when making relevant decisions to maintain competitive parity or limit competition. Since 2020, the COVID-19 epidemic has increased the economic uncertainty and the operating risks of firms. The firms’ managers have been exploring possible pathways to manage and overcome financial distress and crisis. The practical significance of peer effects is to guide managers to respond to the crisis by imitating the excellent firm behavior of peers. Thus, the firm should take steps to increase its imitation capabilities. For example, as the top financial officer of the firm, the CFO should pay greater attention to peers’ activities and behaviors.

Secondly, working capital holdings are negatively correlated with the net profit margin of peer assets, indicating that to obtain higher profit margins, working capital holdings must be reduced, but working capital is closely related to operating cash flow, and the timing and amount of operating cash flow is uncertain, as well as the mismatch between cash inflows and outflows in time, making the firm often have a cash flow gap, which increases the firm’s inability to timely repay debt. This increases the risk that the firm will not be able to pay its debts on time. The solution to this dilemma is to accelerate working capital turnover and support as much operating income with as little working capital as possible in order to keep the firm free from solvency risk.

Thirdly, knowledge flow among peer firms is an important channel to transfer information. Information acquisition not only comes from the financial characteristics of peers, but also from information outside the peer firms. The government has more information advantages than firms, and should establish an effective information disclosure platform to regularly provide true and reliable investment information. At the same time, the government should communicate and exchange investment information policies with firms in a timely manner to enable firms to obtain more comprehensive information, so that firms improve the quality and efficiency of working capital management.

## Data availability statement

The raw data supporting the conclusions of this article will be made available by the authors, without undue reservation.

## Ethics statement

Ethical review and approval was not required for the study on human participants in accordance with the local legislation and institutional requirements. Written informed consent for participation was not required for this study in accordance with the national legislation and the institutional requirements.

## Author contributions

MZ contributed to establishment of the theory, the writing—original draft preparation, and the software. BM helped to analyze the data and editing. YL contributed to the calculations. All authors have read and agreed to the published version of the manuscript.

## Funding

This research was supported by the project of a core course for graduate students of Yunnan Normal University under Grant No. YH2020-C09 and by the project for Case Database-Building of Yunnan Provincial Department of Education.

## Conflict of interest

The authors declare that the research was conducted in the absence of any commercial or financial relationships that could be construed as a potential conflict of interest.

## Publisher’s note

All claims expressed in this article are solely those of the authors and do not necessarily represent those of their affiliated organizations, or those of the publisher, the editors and the reviewers. Any product that may be evaluated in this article, or claim that may be made by its manufacturer, is not guaranteed or endorsed by the publisher.
